# Results of concurrent radio-chemotherapy for the treatment of head and neck squamous cell carcinoma in everyday clinical practice with special reference to early mortality

**DOI:** 10.1186/1471-2407-13-610

**Published:** 2013-12-27

**Authors:** Michael Schlumpf, Claude Fischer, Diana Naehrig, Christoph Rochlitz, Martin Buess

**Affiliations:** 1 Head and Neck Cancer Center, Basel University Hospital, Division of Medical Oncology, Division of Radio-oncology, Division of the ENT Clinic Basel University Hospital, Hebelstrasse 20, CH-4031 Basel, Switzerland; 2 St. Claraspital, Kleinriehenstrasse 30, CH-4016 Basel, Switzerland; 3 Kantonsspital Graubünden, CH-7000 Chur, Switzerland; 4 Sydney Cancer Centre, Royal Prince Alfred Hospital, Camperdown, NSW 2050 Australia

**Keywords:** Chemo-radiotherapy, Head and neck squamous cell carcinoma, Every day practice

## Abstract

**Background:**

Randomized controlled trials have established concurrent chemo-radiotherapy as the preferred treatment option for inoperable local-regionally advanced head and neck squamous cell carcinomas (HNSCCs). Because many patients have multiple co-morbidities and would not fulfill the eligibility criteria of clinical trials, the results need to be re-evaluated in daily clinical practice with special reference to early mortality.

**Methods:**

167 consecutive patients with HNSCC who received concurrent chemo-radiotherapy at the Basel University Hospital between 1988 and 2006 were analyzed retrospectively with a special focus on early deaths and risk factors for an unfavorable outcome.

**Results:**

In our cohort, the 3- and 5-year overall survival rates were 54% and 47%, respectively. The therapy was associated with relevant toxicity and an early mortality rate of 5.4%. Patients dying early were analyzed individually for the cause of death. Patients with elevated white blood cell counts (HR: 2.66 p = 0,016) and vascular co-morbidities (HR: 5.3, p = 0,047) showed significantly worse survival rates. The same factors were associated with a trend toward increased treatment-related mortality. The 3-year survival rate improved from approximately 43% for patients treated before the year 2000 to 65% for patients treated after the year 2000 (Fisher’s exact test p = 0.01).

**Conclusions:**

Although many patients who received concurrent chemo-radiotherapy would not have qualified for clinical trials, the outcome was favorable and has significantly improved in recent years. However the early mortality was slightly worse than what is described in the literature.

## Background

Approximately 60% of patients with head and neck squamous cell carcinomas (HNSCCs) are diagnosed with a locally advanced stage (stages III and IV) [1]. Without treatment, the median survival is less than 4 months [2]. Treatment of advanced-stage HNSCC remains challenging. In the past, radiotherapy alone was the treatment of choice for patients with non-resectable or inoperable local-regional disease. Due to unsatisfactory results, intensified radiotherapy and concurrent radiation and chemotherapy protocols were initiated [3]. In early trials with chemo-radiotherapy, there were no improvements in survival, due to an increase in toxicity and therapeutic abortions; however, radiotherapy in combination with cis-platinum resulted in higher complete remission rates of 65% to 70% [3-5]. In a randomized trial, the 3-year survival rate was significantly higher for patients who obtained concurrent chemo-radiotherapy with cis-platinum [6] than radiotherapy alone. The advantages of this regimen were confirmed by multiple trials [6-10], and several meta-analyses have consistently demonstrated that concurrent chemo-radiotherapy and, in particular, platinum-based concurrent chemo-radiotherapy could improve patient survival by a magnitude of 8 to 11 percent compared to radiotherapy alone [11]. However, the better long-term results come at the price of a higher toxicity.

Today, in the era of targeted therapies, the combination of radiotherapy with cetuximab, a monoclonal antibody against EGFR that is over-expressed in a majority of HNSCC tumors resulted in significantly improved survival rates compared with radiotherapy alone (55% vs. 45% after 3 years) and a better local-regional control [12]. However, despite promising results, the role of cetuximab in therapeutic regimens remains uncertain; as yet, no direct comparisons of this drug to other chemotherapy drugs (such as cis-platinum) exist.

The improved survival rates from concurrent chemo-radiotherapy come at the price of increased therapy-associated morbidity due to myelosuppression, dermatitis, mucositis, and diarrhea. The patients with numerous co-morbidities and poor general health are particularly vulnerable to these toxicities and might therefore be unsuitable for this treatment option [13]. The restrictive inclusion criteria of most trials would exclude many HNSCC patients with co-morbidities due to chronic alcohol and nicotine consumption. Despite representing the majority of patients, evidence-based guidelines in these subsets of patients with co-morbidities are largely nonexistent. Outside of controlled trials, more frequent discontinuations of treatment, delays in the irradiation protocol and reductions in radiation doses occur, which indicate that certain results obtained in trials can only be partially transferred to the clinical practice. Thus, concurrent chemo-radiotherapy cannot be adopted as the standard treatment for all patients with advanced-stage disease [14].

A number of unexpected deaths during or shortly after concurrent chemo-radiotherapy in our clinic prompted this analysis. The aim of our study was to analyze retrospectively the results of concurrent chemo-radiotherapy in a setting outside of a prospective clinical trial at the Basel University Hospital. Early mortality within 30 days of therapy was of special interest. Specifically, our questions were as follows: 1. What are the results of concurrent chemo-radiotherapy in the cohort of locally advanced HNSCC patients treated in the Head and Neck Cancer Center of the Basel University Hospital? 2. How many patients died during or within 30 days of concurrent chemo-radiotherapy? 3. How does this patient population compare to the patients treated within a clinical trial? 4. Are there any predictive factors for early mortality?

## Methods

A total of 167 consecutive patients who were treated at the Interdisciplinary Head and Neck Cancer Center of the University Hospital of Basel were included. All patients had pathologically proven HNSCC. HPV status was not determined. The patients had been evaluated at the interdisciplinary tumor board by experienced specialists of the cancer center prior to treatment with concurrent chemo-radiotherapy. The relevant data were extracted from patient charts. Before treatment, the clinical, laboratory and imaging examinations were performed to determine an accurate TNM stage (TNM 6^th^ edition) [15]. The staging procedure did not include PET scans. During therapy, the patients were assessed at least weekly for signs of toxicity. This retrospective analysis was approved by the ethical committee beider Basel, Switzerland, (EKBB 360/2009).

The following data were assessed: age; sex; performance status (PS); histology; stage; smoking and alcohol consumption history; co-morbidities (e.g. concurrent vascular disease); laboratory values (*i.e.*, hemoglobin levels, leukocyte counts, and serum albumin levels); duration and type of chemotherapy and radiotherapy; radiological response according to WHO criteria and overall survival time.

### Statistical analyses

To determine whether patient outcome has improved over time, patients were divided into two cohorts of either before or after the year 2000.

The variables related to the patients, diseases, and treatments, were compared among cohorts using the chi-square test for discrete variables and the Mann- Whitney *U* test for continuous variables. The probabilities of survival were calculated using the Kaplan-Meier estimator. The log-rank test was used for comparing groups. The following variables were analyzed for association with survival:

patient characteristics (age, sex, smoking history, and weight loss); disease characteristics (disease stage and histology) and laboratory values (white blood cell count, hemoglobin, and albumin).

The relative risk was calculated with the MedCalc software; RR = a/(a + b)/c/(c + d).

## Results

### Patient characteristics

A total of 167 consecutive patients with HNSCC receiving concurrent chemo-radiotherapy between 1988 and 2006 at the Head and Neck Cancer Center of the Basel University Hospital were analyzed retrospectively. The median age was 57 years (25–82 years); 80% of the patients were male. Nicotine and/or alcohol consumption was commonly reported. Most tumors were located in the piriform sinus (n = 32), the base of the tongue (n = 27), the tonsils (n = 24) and in the hypopharynx (n = 20). In 117 patients, lymph node metastases were detected: 76 on the ipsi-lateral side, 39 bilaterally and in 2 patients, the nodal metastasis occurred only contra-laterally. Metastases in the lung were described in one patient; in all other patients, no distant metastases were documented. The staging according to the American Joint Committee on Cancer (AJCC) resulted in 1 patient with stage I cancer, 8 with stage II, 27 with stage III and 129 with stage IV. In the patients with stage I-III carcinomas, primary surgery was not performed due to considerations regarding functional or cosmetic organ preservation. Eight of the 167 patients showed a synchronous secondary carcinoma. Before treatment, 36 patients (44%) had concurrent vascular disease (n = 82; for 85 patients, there were no data available). In addition, 31 patients (35%) had hemoglobin values below the normal range (normal range: 135–175 g/l) (n = 79), 13 patients (16%) showed increased leukocytes counts (normal range: 4.5-11.5 g/l) (n = 79) and 33 patients (45%) had albumin levels below the normal range (normal range: 37–51 g/l) (n = 74).

A total of 88% of patients started concurrent chemo-radiotherapy within the first three months after diagnosis. Cis-platinum was the primary treatment and was frequently used as a single agent in combination with radiotherapy. Among patients treated with radiotherapy and combination chemotherapy regimens, cis-platinum/5-FU and carbo-platinum/5-FU were most frequent. Several patients were treated in a pilot study with irradiation in combination with cis-platinum and gefitinib, a tyrosine-kinase EGFR inhibitor. The doses of radiotherapy were above 70 Gy for most patients; 23% of patients received a total dose of 60–70 Gy, and 14% received less than 60 Gy. The duration of treatment was in the standard range, typically lasting 40 to 60 days.

### Results of the concurrent chemo-radiotherapy

Of the 167 patients, 98 (59%) showed a complete response, 23 (14%) had a partial remission, eight (5%) showed no change in disease status, six (3%) showed tumor progression, nine (5%) died during therapy, and for 23 patients (14%), the response data were missing.

**Figure 1:**
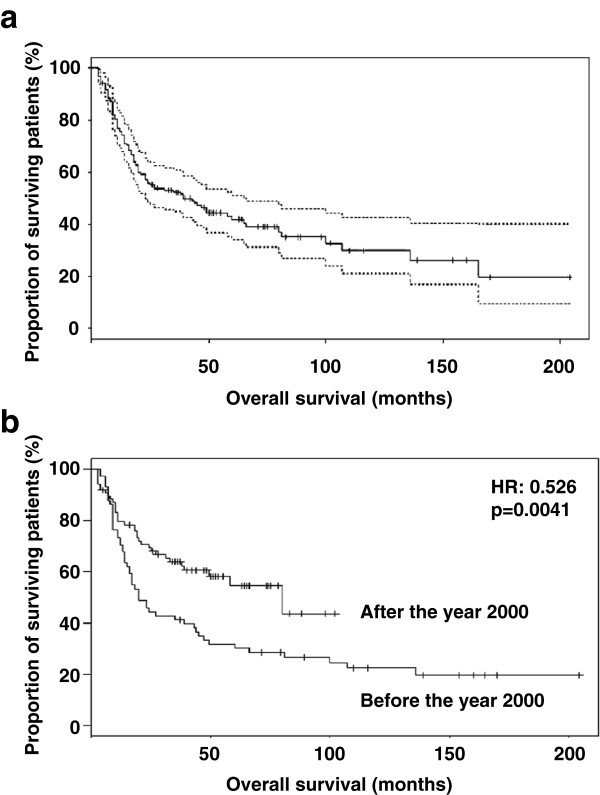
**Kaplan-Meier plots of overall-survival estimates. (a)** All patients treated with concurrent chemo-radiotherapy at the Head and Neck Cancer Center of the Basel University Hospital. The 95% confidence interval is indicated by dotted lines **(b)**. The patients treated after the year 2000 (n = 72) showed an improved survival rate with a hazard ratio of 0.526 (p = 0.0041) compared to the patients treated before the year 2000 (n = 85).

One year after diagnosis, there were 125 patients (75%) alive, three (2%) died in the absence of a tumor and ten (6%) died from the tumor. For six patients (4%), there was no information available except that they did not die while under therapy. Nine patients (5%) died during or within 3 weeks of therapy, and there was no information available for 14 patients (8%; n = 167). The 3-year overall survival rate was 54%, and the 5-year survival rate was 47%. The median overall survival was 44 months (range 3–204 months; Figure 1a). In recent years, since the year 2000, concurrent chemo-radiotherapy has improved. The median follow-up of patients who were treated after the year 2000 was 3 years, while the patients treated before the year 2000 had a longer follow-up time. The 3-year survival rate has risen from 43% in the years before 2000 to over 65% in the years after 2000 (two-sided Fisher’s exact test, p = 0.01). The patients who were treated before the year 2000 showed a median survival of 20 months, while patients treated after the year 2000 lived longer, and the median survival was not reached after 36 months (Figure 1b).

### Therapy-associated toxicity

Under therapy, approximately half of the patients suffered from severe stomatitis > grade 2. The pretreatment hemoglobin values were below the normal range (men < 140 g/l, women < 120 g/l) in 28 out of 79 patients (36%; for 88 patients, there were no data available), and 76 out of 84 patients (90%) developed anemia during the therapy. Also, 70 out of 72 patients (97%) developed hypoalbuminemia (< 37 g/l) during therapy. Neutropenia occurred in 14 out of 53 patients (28%); 11 of these patients (20%) developed febrile neutropenia. Nausea and vomiting were documented in 21 of 56 patients (37%), and 79 out of 81 patients (97%) presented with stomatitis > grade II.

#### Early mortality during concurrent therapy

Nine patients died during or within three weeks of chemo-radiotherapy, resulting in an early mortality rate of 5.4%. Three of these patients were female; the median age was 59 years. The patients who died under therapy had consumed an average of 54 pack-years of nicotine, and alcohol consumption was reported for eight. The patients dying under therapy were analyzed individually for the cause of death; the autopsy results are summarized in Table 1. One patient died from neutropenic infection, a side effect of chemotherapy known to be potentially fatal. In the other eight patients who died during therapy, we did not find any obvious well-described complication of concurrent chemo-radiotherapy.

**Table 1:** A summary of the causes of death while under therapy

	**Cause of death:**
Case 1	heart failure
Case 2	heart failure
Case 3	aspiration pneumonia
Case 4	liver failure
Case 5	Unknown (no autopsy)
Case 6	heart failure
Case 7	myocardial infarction
Case 8	protracted shock
Case 9	ischemia of the small bowel

#### The prognostic impact of hemoglobin, albumin, white blood cell counts, vascular co-morbidities, and alcohol and nicotine consumption

**Figure 2:**
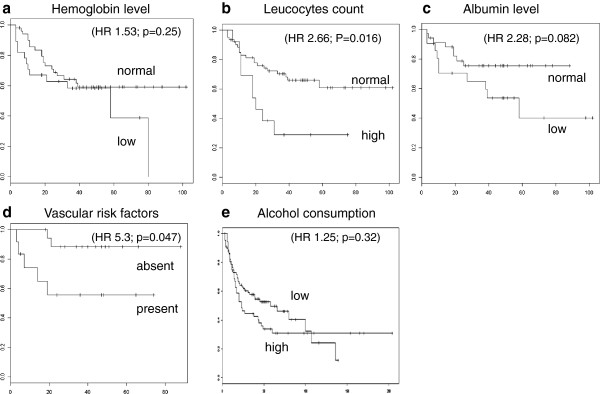
**The prognostic impact of hemoglobin levels, leukocyte counts, albumin levels, vascular co-morbidities and alcohol consumption.** The patient overall survival depends on **(a)** hemoglobin levels (HR 1.53; p = 0.25), **(b)** leukocyte counts (HR 2.26; P = 0.016), **(c)** pre-therapeutic albumin levels (HR 2.28; p = 0.082), **(d)** vascular risk factors (HR 5.3; p = 0.047) and **(e)** alcohol consumption (HR 1.25; p = 0.32).

To determine whether patients who died during therapy differed significantly from patients benefiting from treatment, we compared the following parameters in the two populations: pre-therapeutic hemoglobin levels, white blood cell counts, albumin levels, the presence of vascular co-morbidities, and nicotine and alcohol consumption. The first question was whether these factors have a prognostic significance. In patients with elevated white blood cell counts before treatment, the median survival was 20 months compared to 39 months for patients with normal white blood cell counts; this result indicated a significant difference (n = 74, HR = 2.66, P = 0,016). Anemia before initiating therapy reduced the median survival to 30.5 months compared to 37 months in patients with normal hemoglobin levels (n = 79, HR = 1.53, p = 0.25). The patients with hypoalbuminemia before therapy showed a median overall survival of 38 months compared to 40 months in patients with normal albumin levels (n = 56, HR = 2.28, p = 0,082). The patients with vascular co-morbidities prior to therapy showed a median survival of 21.5 months compared to 42 months (n = 30, HR = 5.3, p = 0.047). The patients with alcohol use before starting treatment showed a 24-month median survival compared to those without alcohol abuse who had a median survival of 48 months, which was not a significant difference (n = 138, HR = 1.25, p = 0.32) (Figure 2).

#### A description of risk factors associated with death while under therapy

To select patients for chemo-radiotherapy, we were interested in factors predicting an early death while under therapy. To this end, we analyzed whether vascular co-morbidity, alcohol, nicotine, hemoglobin and albumin levels distinguished patients who died under treatment from patients who benefited with a long survival. Vascular co-morbidities were present in 75% of the deceased patients compared to 40% of the remaining patients (p = 0.09). In addition, 90% of the patients who died under therapy consumed alcohol, whereas only 60% of the patients who benefitted from therapy consumed alcohol (p = 0.11). Furthermore, 90% of the patients who died under therapy smoked on average 54 pack-years. Before the initiation of therapy, one third of the deceased later showed anemia, whereas the remaining patients were affected only by 33% (p = 0.09). The albumin levels were low in 60% of the patients who died under therapy and in 40% of the remaining patients (p = 0.29). Therefore, none of these factors was significantly associated with death while under therapy. The data are summarized in Table 2. High alcohol consumption showed a trend toward predicting therapy-associated mortality.

**Table 2:** The potential risk factors and their association with death under concurrent chemo-radiotherapy

**Predictive factors for early death under therapy**				
	**Dead**	**Alive**	**Relative risk**	**p-value**
**Vascular co-morbidities**				
**Present**	6	30	3.83	0.09
**Absent**	2	44		
**No data**	1	84		
**Alcohol consumption**				
**Yes**	8	80	5.27	0.11
**No**	1	57		
**No data**	21			
**Nicotine consumption**				
**Yes**	8	86	6.2	0.081
**No**	1	72		
**Hemoglobin**				
**Below normal range**	6	25	3.09	0.09
**Normal**	3	45		
**No data**	0	88		
**Albumin**				
**Below normal range**	5	28	2.07	0.29
**Normal**	3	38		
**No data**	1	92		

## Discussion

In our institution, the 3-year survival rate of locally advanced HNSCC after concurrent chemo-radiotherapy was 54%. This result is comparable to the results obtained within the relevant clinical trials (Table 3) [6-10,12,16-18]. Thus, it appears that the therapeutic results of our patient cohort outside of a clinical trial lie within the average range of the results from the published clinical trials. Importantly, in recent years, the therapeutic results of our institution have improved significantly. The patients treated after the year 2000 had a survival rate of 65%, and those treated before the year 2000 showed a survival rate of 43% at 3 years (HR 0.526, p = 0.0041). Today, the survival rate of 65% at 3 years compares well with the data from published clinical trials.

**Table 3:** A cross-study comparison of long term survival data and early death rates of the patients with HNSCC from Basel and patients with HNSCC who had been treated within published clinical trials with concurrent chemo-radiotherapy

**Study**	**Year**	**3-OS (in %)**	**5-OS (in %)**	**Early mortality (patients)**
**Bonner et. al.**	2006	55	40*	11/211 (5%)
**Huguenin et. al.**	2004	59°	46	0/112 (0%)
**Calais et. al.**	1999	51	-	1/109 (1%)
**Merlano et. al.**	1996	38*	24	not reported
**Adelstein et. al.**	2000	37	28*	6/96 (6%)
**Wendt et. al.**	1998	49	-	4/256 (2%)
**Brizel et. al.**	1998	55	48*	1/56 (2%)
**Al-Sarraf et. al.**	1998	78	68*	1/78 (1%)
**Data from Basel**	1994-2007	54	47	9/167 (5%)
**- Therapy before 2000**		43	33	
**- Therapy after 2000**		65	61	

°Survival after 2.5 years.

*estimated value.

A main cause for the better survival rates in the cohort treated after the year 2000 might be the improved radiotherapy technique with the introduction of 3D-CRT (3-dimensional conformal radiotherapy) with isocentric CT-planning at our institute in the year 2001 with maximum dose levels of up to 74 Gy in the tumor area replacing the 2D-SSD technique. The dose prescription pertains to the appropriate PTV area: 74 Gy to the primary tumor and macroscopically positive lymph nodes (boost area). Per definition the CTV area covers all macroscopic disease (tumor and positive lymphnodes). The CTV is the area at risk of microscopic involvement. The PTV is a safety margin that is added to the CTV to allow for set-up errors and movement. In general the ipsilateral nodal drainage areas were treated with 56 Gy and the contralateral drainage areas with 50 Gy.

None of the patients was treated with IMRT (intensity modulated ratiotherapy). Other potential reasons for improvement in outcome such as chemotherapy schedules and doses could not be evaluated in this retrospective study. Furthermore, the results with respect to overall survival appear to support the selection strategy applied by the interdisciplinary head and neck cancer center team. Of note, outside of a setting with an experienced interdisciplinary team, the results might differ significantly.

### Comparison of therapy-associated mortality

Nine out of 167 patients died during or within 30 days after completion of therapy, an observation that led to the initiation of this retrospective analysis. Our hypothesis was that the rate of early mortality might be too high in our institution. Examining the above mentioned trials with respect to early death rates, we obtained the information as shown in Table 3. These results indicate that compared to these trials, our cohort ranged within the 95-99% worst values. This is not surprising in a patient population outside of a clinical trial for which usually less rigid criteria are applied to exclude a patient from the treatment.

### Comparison of the eligibility criteria

To identify differences between our HNSCC population and the patients treated within the relevant clinical trials, the eligibility criteria have been analyzed. In the trial by Bernier et al. [18], patients with anemia (<110 g/l) were excluded from the trial. This restriction indicates that four out of nine patients who died under therapy in our cohort would have been excluded from this treatment approach. However, 25 of 70 patients (35%) who survived the treatment would have also been excluded and would have missed the benefits of chemo-radiotherapy. In the same trial, the age limit was set to 70 years; 17 of our patients would not have qualified due to the age restriction and one of the patients over 70 years died under therapy. In total, five out of our nine patients who died under treatment would not have met the 2 abovementioned inclusion criteria and therefore would not have been eligible for the treatment according to the trial. In the study by Bonner et al. [12], normal hematological, hepatic and renal function were a prerequisite for the treatment, which would have excluded 50 of our patients. According to this study, 6 out of the 9 patients who died under treatment would not have met the eligibility criteria and thus would have been excluded. These comparisons show that our patients represent a different population from that of the clinical trials and are characterized by increased co-morbidities and risk factors, thereby explaining the larger treatment-associated mortality. Overall, the eligibility criteria within the clinical trial protocols were safer; however, they also excluded many patients who successfully completed the treatment and appear to have benefited from this therapeutic approach. Although patient selection was based on the decisions of an experienced interdisciplinary team, a higher number of early deaths during therapy was observed, supporting the notion that a checklist of inclusion and exclusion criteria, as used in the clinical trial setting, would be a safe way to minimize early deaths. Despite the fact that many patients who were treated at University Hospital in Basel would never have fulfilled the inclusion criteria of a clinical trial, the 3-year survival data were comparable to the results from the published clinical trials in a patient collective that was considerably larger than the highly selected trial population. This finding indicates that the rigid selection of a clinical trial also eliminates many patients who derive a benefit from the therapy. Whether the patients with early deaths died due to treatment complications or due to the disease or co-morbidities cannot be definitely decided in a single-arm study. The question remains of whether a higher early mortality risk is considered acceptable in view of the favorable overall survival results of a cohort with less stringent patient selection.

Unfortunately, among all of the factors analyzed for prognostic value, none was significantly associated with early death, and we could not identify any predictive marker for therapy-associated mortality.

## Conclusions

Despite having a rather high early mortality rate, the overall outcome of our cohort of HNSCC patients treated with concurrent chemo-radiotherapy is favorable and has improved in recent years. Because our study did not reveal significant predictive markers for early fatal outcomes, we suggest for the moment not to withhold concurrent chemo-radiotherapy based only on such a marker. However, these risk factors should give rise to greater caution and be discussed with patients before therapy. In conclusion, the analyses of our data confirmed that chemo-radiotherapy performed by an experienced team can reach similar results in a population outside of a clinical trial and that considerable progress has been made with the use of concurrent chemo-radiotherapy to treat HNSCC over the last decade. An experienced team is necessary to perform the treatments and manage the therapy-associated toxicities.

## Competing interests

The authors declare that they have no competing interests.

## Authors’ contributions

MS, CR, MB designed the study. MS,CF, DN provided and collected the patients data. MS, MB analyzed and interpreted the data. MS, MB wrote the paper. MS, CF, DN, CR and MB approved the paper. All authors read and approved the final manuscript.

## Pre-publication history

The pre-publication history for this paper can be accessed here:

http://www.biomedcentral.com/1471-2407/13/610/prepub
